# INTENTION TO USE HOSPICE CARE FOR FAMILY AMONG OLDER RURAL BLACK AMERICANS IN ALABAMA

**DOI:** 10.1093/geroni/igad104.2507

**Published:** 2023-12-21

**Authors:** Ellen Csikai, Hee Yun Lee

**Affiliations:** University of Alabama, Tuscaloosa, Alabama, United States; The University of Alabama, Tuscaloosa, Alabama, United States

## Abstract

Hospice usage overall continues to rise however, remains low among people of color, including by Black/African Americans. In 2020, 50.8% of White Medicare decedents and only 35.5% of Black decedents used hospice (NHPCO, 2022). Often reasons are connected to poor knowledge of and access to hospice resources that provide holistic support to those near end of life and their caregivers (Adrien, 2017). Little is known about the intention of older rural Black Americans’ to use hospice when caring for family members at home. In this study, older African Americans in Black Belt communities in Alabama were recruited to participate in a survey that addressed health literacy of problems with high prevalence in this rural area (diabetes, cardiovascular disease, stroke and cancer) (CDC Interactive Atlas, 2022). Guided by the Andersen’s Behavioral Model of Health Services Use, regression analysis was conducted to identify predisposing, enabling, and need factors influential in older rural African Americans’ intention to use hospice for family members. Among non-caregivers, who had heard of hospice (n=118 of 180), education, gathering with friends and social club participation, and PHQ-9 were found influential in predicting intention to use hospice for family members. However, among caregivers (n=107), female gender, education, health literacy, gathering with friends, social club participation, and PHQ-9 were influential. Results point to interventions to bolster health literacy regarding hospice among older rural Black Americans and that social clubs and other gathering places could be targeted with educational programs to increase intention to use hospice care for family members.

